# Nociceptor activity induces nonionotropic NMDA receptor signaling to enable spinal reconsolidation and reverse pathological pain

**DOI:** 10.1126/sciadv.adg2819

**Published:** 2023-05-19

**Authors:** Hantao Zhang, Luis D. Rodriguez-Hernandez, Abigail J. D’Souza, David He, Maham Zain, Samuel W. Fung, Laura A. Bennett, Robert P. Bonin

**Affiliations:** ^1^ Department of Pharmaceutical Sciences, Leslie Dan Faculty of Pharmacy, University of Toronto, Toronto, Ontario, Canada.; ^2^ Department of Anesthesia, Temerty Faculty of Medicine, University of Toronto, Toronto, Ontario, Canada.; ^3^ Department of Cell and Systems Biology, University of Toronto, Toronto, Ontario, Canada.; ^4^ University of Toronto Centre for the Study of Pain, University of Toronto, Toronto, Ontario, Canada.

## Abstract

Chronic, pathological pain is a highly debilitating condition that can arise and be maintained through central sensitization. Central sensitization shares mechanistic and phenotypic parallels with memory formation. In a sensory model of memory reconsolidation, plastic changes underlying pain hypersensitivity can be dynamically regulated and reversed following the reactivation of sensitized sensory pathways. However, the mechanisms by which synaptic reactivation induces destabilization of the spinal “pain engram” are unclear. We identified nonionotropic *N*-methyl-d-aspartate receptor (NI-NMDAR) signaling as necessary and sufficient for the reactive destabilization of dorsal horn long-term potentiation and the reversal of mechanical sensitization associated with central sensitization. NI-NMDAR signaling engaged directly or through the reactivation of sensitized sensory networks was associated with the degradation of excitatory postsynaptic proteins. Our findings identify NI-NMDAR signaling as a putative synaptic mechanism by which engrams are destabilized in reconsolidation and as a potential means of treating underlying causes of chronic pain.

## Teaser

Nonionotropic NMDA receptor signaling offers a potential treatment for chronic pain by destabilizing spinal pain memories.

## INTRODUCTION

Many treatments for pain, such as opioids, dampen neuronal activity to suppress nociceptive processing but do not address underlying causes of pain. Central sensitization or nociplastic pain represents particularly intractable sources of chronic pain, as these “pain memory traces” (*1*, *2*) can persist long after being triggered by an insult or injury (*3*). Memory retrieval can destabilize a previously consolidated memory (i.e., reactive destabilization) and then subsequently induce protein synthesis-dependent restabilization (reconsolidation) of the engram to enable memory updating and strengthening (*4*–*6*). Specifically, blocking memory reconsolidation has inspired treatments for psychological disorders, such as substance use disorder, posttraumatic stress disorder, anxiety disorder, and beyond (*7*, *8*). At the cellular level, reactive destabilization can also trigger two distinct processes of protein synthesis and protein degradation that underlie synaptic plasticity (*6*, *9*, *10*). The intracellular mechanisms of synaptic potentiation following reactive destabilization have been well characterized and linked with reconsolidation (*10*, *11*). However, less is known about which signaling cascades directly contribute to the synaptic depotentiation enabled by reactive destabilization that has therapeutic potential for pathological pain.

Our previous work has demonstrated that spinal pain memory traces can be modified in a process analogous to memory reconsolidation (*12*). We reported that the reactivation of sensitized nociceptive pathways could similarly trigger the destabilization of plastic changes in the spinal cord. Preventing restabilization through protein synthesis inhibition led to the reversal of pain hypersensitivity and dorsal horn long-term potentiation (LTP). These findings suggested that reactive destabilization may be an effective approach to treating central changes contributing to pathological pain. In addition, we showed that reactive destabilization in spinal nociceptive networks requires the activation of *N*-methyl-d-aspartate (NMDA) receptors (NMDARs) (*12*). NMDAR activity is a primary means by which sensitization and synaptic potentiation are initiated in spinal dorsal horn (SDH) nociceptive networks (*13*, *14*). Recent work has indicated that NMDARs can also signal in a noncanonical, nonionotropic manner to reduce synaptic efficacy (*15*–*18*). In these studies, nonionotropic activation of the NMDAR was induced by glutamate binding to the receptor in the absence of glycine binding to the coagonist site or via blockade of the channel pore. The opposing effects of ionotropic and nonionotropic NMDAR (NI-NMDAR) activity may bidirectionally modulate spinal plasticity following activation of nociceptive networks. We therefore sought to examine whether NI-NMDAR signaling initiates the depotentiation process during reactive destabilization.

## RESULTS

### Glutamate, not glycine, binding to NMDAR is necessary for reactive destabilization and reversal of hyperalgesia

**Fig. 1.**
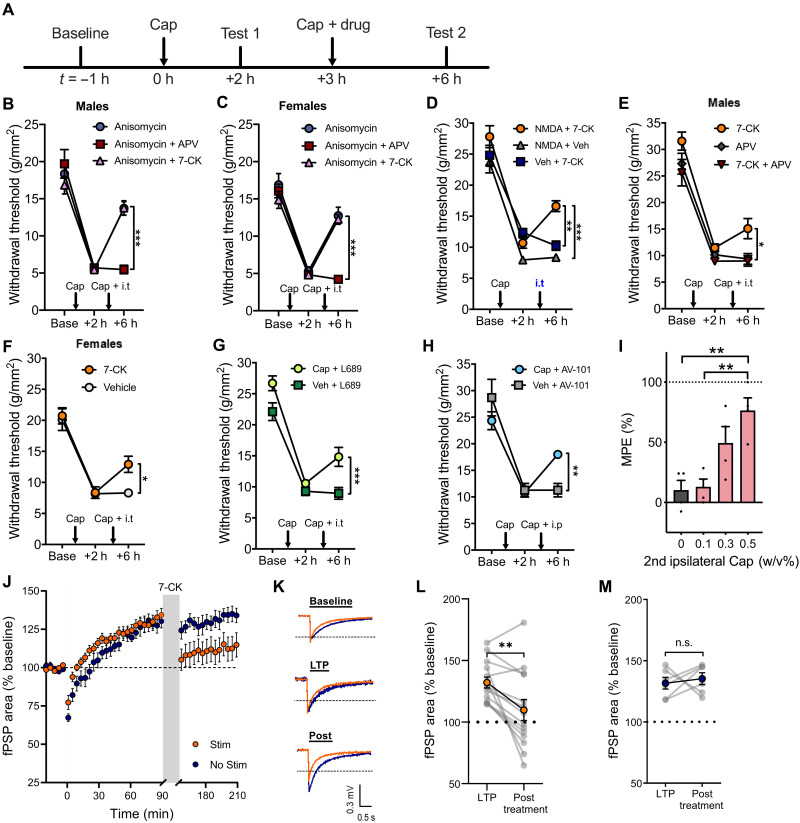
NI-NMDAR activity directly contributes to the reversal of hyperalgesia and dorsal horn LTP following the reactivation of sensitized pain pathways. (**A**) Timeline of experimental protocol. (**B** and **C**) Changes in mechanical withdrawal thresholds induced by intraplantar injection of capsaicin (Cap), followed by a second ipsilateral intraplantar injection of capsaicin and intrathecal injection of anisomycin alone, anisomycin coadministered with APV (NMDAR glutamate site antagonist) or 7-CK (NMDAR glycine site antagonist) in (B) male and (C) female mice. Injection times are indicated by arrows. (**D**) Capsaicin-induced hyperalgesia was followed by intrathecal injection of NMDA + 7-CK for selective activation of NI-NMDAR signaling. 7-CK alone (intrathecal injection) or NMDA alone (intrathecal injection) did not reverse mechanical hypersensitivity. (**E** and **F**) NI-NMDAR signaling was activated by a second ipsilateral intraplantar injection of capsaicin and intrathecal injection of 7-CK, resulting in the reversal capsaicin-induced hyperalgesia in both (E) male and (F) female mice. This reversal was not observed when 7-CK was coadministered with APV. (**G** and **H**) Capsaicin-induced hyperalgesia was followed by a second ipsilateral intraplantar injection of capsaicin or vehicle (Veh), coupled with (G) intrathecal injection of L-689,560 (L689) or (H) intraperitoneal injection of AV-101. (**I**) AV-101 reversal of hyperalgesia using different doses of capsaicin during the second ipsilateral intraplantar injection expressed as percentage of maximum possible effect (MPE). (**J**) LTP of SDH field postsynaptic potentials (fPSPs) was followed by bath application of 7-CK from 90 to 150 min with or without electrical stimulation of dorsal roots. (**K**) Representative traces of fPSPs recorded at baseline, 90 min (LTP) and 210 min (post). (**L** and **M**) Summary data of changes in fPSP area before (LTP) and after (posttreatment) 7-CK application (L) with or (M) without dorsal root stimulation. Data are means ± SEM. Not significant (n.s.), *P* > 0.05; *P < 0.05; **P < 0.01; ***P < 0.001.

We first examined whether glutamate or glycine binding to the NMDAR is necessary for reactive destabilization and reversal of hyperalgesia using the NMDAR glutamate binding site antagonist d,l-2-amino-5-phosphonovaleric acid [APV; 250 μM, intrathecally (i.t.)] or the NMDAR glycine site antagonist 7-chlorokynurenic acid (7-CK; 250 μM, i.t.). Mechanical sensitization was induced by intraplantar injection of capsaicin (5 μl, 0.5%, w/v) in mice. Three hours after the first capsaicin injection, reactivation of the sensitized pathways was triggered by a second intraplantar capsaicin injection at the site of the first injection (Fig. 1A). Consistent with previous findings (*12*), when the second injection of capsaicin was paired with spinal administration of the protein synthesis inhibitor, anisomycin (47 mM, i.t.), we observed a significant reversal of hyperalgesia in both sexes (Fig. 1, B and C). Unexpectedly, this reversal of hyperalgesia was only prevented when APV but not 7-CK was intrathecally coadministered with anisomycin (Fig. 1, B and C). These findings indicate that activation of NMDAR through glutamate binding, not glycine, is necessary for reactive destabilization. Our results also suggest a potential role of NI-NMDAR signaling in reactive destabilization. Notably, when anisomycin and 7-CK were coadministered, there was no additive effect on the reversal of hyperalgesia (Fig. 1, B and C). The lack of additive effects suggests that the two processes share or have overlapping mechanisms.

### NI-NMDAR signaling initiated by nociceptor activity reverses hyperalgesia

Since blockade of the NMDAR glycine site can permit signaling and modulation of plasticity via NI-NMDAR signaling (*15*–*18*), we next examined whether NI-NMDAR activity is sufficient to reverse hyperalgesia and spinal plasticity associated with pathological pain. Mechanical sensitization was again induced by intraplantar injection of capsaicin in mice. When 7-CK (250 μM, i.t.) was intrathecally coadministered with NMDA (75 μM, i.t.) to directly engage NI-NMDAR signaling, we observed a significant reduction in hyperalgesia (Fig. 1D). Notably, intrathecal administration of either NMDA or 7-CK alone did not have any effect on hyperalgesia.

We further observed that intrathecal 7-CK can similarly reverse hyperalgesia in both sexes when paired with a second intraplantar capsaicin injection used to resensitize nociceptive networks (Fig. 1, E and F). Crucially, this reversal of hyperalgesia was no longer observed when 7-CK was coadministered with APV, suggesting that NMDAR activation via glutamate binding is necessary for the reversal of hyperalgesia (Fig. 1E). Comparably, we found that another potent glycine site antagonist L-689,560 (50 μM, i.t.; Fig. 1G) or intraperitoneal injection of the blood-brain barrier permeable prodrug of 7-CK, AV-101 [400 mg/kg, intraperitoneally (i.p.)] (*19*, *20*) reversed hyperalgesia when paired with a second injection of capsaicin (Fig. 1H). The degree to which NI-NMDAR signaling reversed hyperalgesia varied with the concentration of the second intraplantar capsaicin injection but had no effect when administered without readministration of capsaicin (Fig. 1I). 7-CK administration did not affect mechanical sensitivity in naïve animals (fig. S1A). 7-CK also had no effect on the initial sensitization of nociceptive pathways by the first capsaicin injection (fig. S1B), indicating that NI-NMDAR signaling does not generally suppress pain processing. This reversal of hyperalgesia was also independent from GluK1 kainate receptors or metabotropic glutamate receptors mGluR5, which have been previously shown to modulate spinal plasticity (fig. S1C) (*21*, *22*). Together, these results further demonstrate that NI-NMDAR signaling is sufficient to reverse hyperalgesia and can be initiated by nociceptor activity.

### Activity-dependent modulation of synaptic plasticity in the SDH is mediated by NI-NMDAR signaling

LTP in the spinal cord dorsal horn can contribute to central sensitization and enhanced pain processing (*23*). Because NI-NMDAR signaling can lead to a reduction in synaptic efficacy and synaptic loss in vitro (*15*, *16*), we next tested whether NI-NMDAR activity can also reverse LTP in ex vivo spinal cord explants. After the induction of dorsal horn LTP, dorsal roots were electrically stimulated to reactivate sensitized pathways during bath application of 7-CK (100 μM); this led to a long-lasting reduction in the magnitude of extracellularly recorded field postsynaptic potentials (fPSPs; Fig. 1, J to L). Bath application of 7-CK without dorsal root stimulation did not change the magnitude of LTP (Fig. 1M), indicating that NI-NMDAR modulation of synaptic plasticity was activity dependent and required the activation of previously sensitized pathways. Activity-dependent reversal of LTP was also achieved using the potent glycine site antagonist, L-689,560 (10 μM; fig. S2, A to C). However, inhibition of NMDAR activity with APV (100 μM) did not reverse LTP (fig. S2, D to F), further confirming that glutamate binding to NMDAR was necessary for depotentiation in the SDH. 7-CK did not affect fPSPs magnitude without prior induction of dorsal horn LTP (fig. S3, A to D), paralleling our observations from behavioral experiments that NI-NMDAR signaling induces selective depotentiation of sensitized nociceptive networks rather than nonspecific reduction in synaptic strength. The reversal of LTP by NI-NMDAR activity was observed in spinal cord explants from mice of both sexes (fig. S4, A to C) and explants from both C57BL/6 N and CD-1 mice (fig. S4, D to F), suggesting that NI-NMDAR signaling is a conserved mechanism that leads to depotentiation in the SDH across strain and sex.

### NI-NMDAR activity elicits long-lasting analgesic effects

**Fig. 2.**
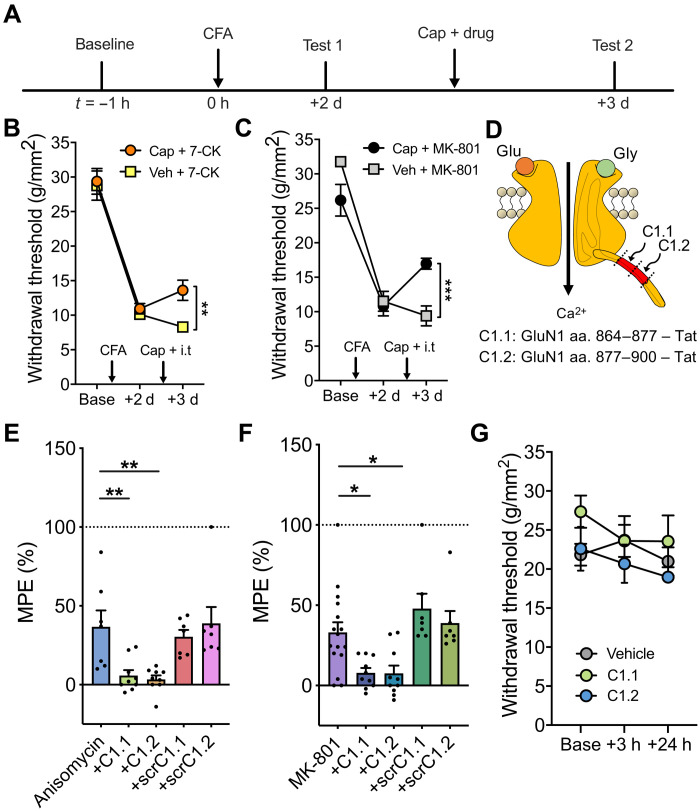
NI-NMDAR activity and reactive destabilization produce long-lasting analgesic effects that are both dependent on GluN1 C-terminal interactions. (**A**) Timeline of experimental protocol. (**B** and **C**) Changes in mechanical withdrawal thresholds induced by intraplantar CFA followed by a second ipsilateral intraplantar injection of capsaicin or vehicle and intrathecal injection of (B) 7-CK (NMDAR glycine site antagonist) or (C) MK-801 (NMDAR pore blocker). (**D**) Schematic diagram of GluN1 C-terminal target sites for C1.1 and C1.2 mimetic peptides. aa., amino acid. (**E**) Summary of antihyperalgesia induced by intrathecal injection of anisomycin alone, anisomycin + mimetic Tat peptides (C1.1 or C1.2), or anisomycin + scrambled peptide controls (scrC1.1 or scrC1.2), expressed as percentage of MPE. (**F**) Summary of antihyperalgesia induced by intrathecal injection of MK-801 alone, MK-801 + mimetic Tat peptides (C1.1 or C1.2), or MK-801 + scrambled peptide controls (scrC1.1 or scrC1.2), expressed as percentage of MPE. (**G**) Intrathecal injection of vehicle, C1.1, or C1.2 mimetic peptides had no effect on the withdrawal threshold of naïve C57BL/6 N mice. Data are means ± SEM. *P < 0.05; **P < 0.01; ***P < 0.001.

We next examined the ability of NI-NMDAR activity to cause lasting reversal of hyperalgesia in the complete Freund’s adjuvant (CFA) model of inflammatory hyperalgesia, which induces mechanical sensitization lasting up to 2 weeks. NI-NMDAR signaling was induced during the phase of peak hyperalgesia, 2 days after intraplantar injection of CFA (10 μl) (*12*). NI-NMDAR signaling was induced by intrathecal administration of 7-CK (250 μM, i.t.) or MK-801 (2 mM, i.t.; an NMDAR pore blocker), followed by resensitization via ipsilateral intraplantar injection of capsaicin to activate peripheral nociceptors. Mechanical sensitivity was tested 24 hours after drug administration to examine whether NI-NMDAR signaling can persistently reverse sensitization (Fig. 2A). Our results indicate that CFA-induced hyperalgesia was reduced by both 7-CK (Fig. 2B) and MK-801 (Fig. 2C) when paired with intraplantar capsaicin. Furthermore, NI-NMDAR signaling produced a lasting reduction of CFA-induced hyperalgesia that persisted until the resolution of hyperalgesia (fig. S5, A and B). We observed a similarly persistent reduction in hyperalgesia when NI-NMDAR signaling was induced 7 days after intraplantar CFA injection (fig. S5, C and D). Collectively, these results indicate that NI-NMDAR activation can elicit a long-lasting analgesic effect and significantly enhance recovery from hyperalgesia at various time points after pain onset.

### GluN1 C-terminal interactions are necessary for the reversal of hyperalgesia produced by anisomycin and MK-801

The reversal of sensitization by activity-induced NI-NMDAR signaling bears strong parallels to reactive destabilization or our sensory model of “reconsolidation” previously reported (*12*). However, the mechanisms by which reactive destabilization reverses hyperalgesia have not yet been characterized. We therefore examined whether synaptic depotentiation generated by reactive destabilization requires NI-NMDAR signaling. Previous research showed that intracellular delivery of antibodies targeting the C-terminal tail of the NMDAR prevented NI-NMDAR signaling and associated synaptic depression, indicating that signaling via the C-terminal tail of the GluN1 subunit is crucial for coordinating downstream effects of NI-NMDAR signaling (*18*). We therefore developed membrane permeable transactivator of transcription (Tat)–conjugated peptides that mimic regions of the GluN1 C-terminal tail with the aim of interfering with protein-protein interactions necessary for intracellular NI-NMDAR signaling (Fig. 2D) (*24*). These peptides were coadministered intrathecally (500 μM, i.t.) with either anisomycin (47 mM, i.t.) or MK-801 (2 mM, i.t.) before resensitization in CFA-treated mice. The reversal of hyperalgesia mediated by anisomycin was prevented by both mimetic peptides C1.1 and C1.2 that mimicked separate segments of the C1 domain of the C-terminal tail of GluN1 (Fig. 2E). Similarly, these mimetic peptides also blocked the reversal of hyperalgesia by NI-NMDAR signaling (Fig. 2F). In both cases, membrane permeable scrambled peptide controls of C1.1 or C1.2 had no effect on anisomycin or MK-801–mediated reversal of hyperalgesia. Notably, C1.1 and C1.2 mimetic peptides also had no effect on mechanical sensitivity in naïve mice (Fig. 2G). These results support previous work showing that the C-terminal tail of the GluN1 subunit, particularly the C1-domain, is involved in NI-NMDAR signaling. Our findings further indicate that NI-NMDAR signaling is necessary for the reversal of pain hypersensitivity by reactive destabilization.

### Anisomycin and 7-CK induce the reversal of hyperalgesia and dorsal horn LTP via a protein phosphatase 1–dependent mechanism

**Fig. 3.**
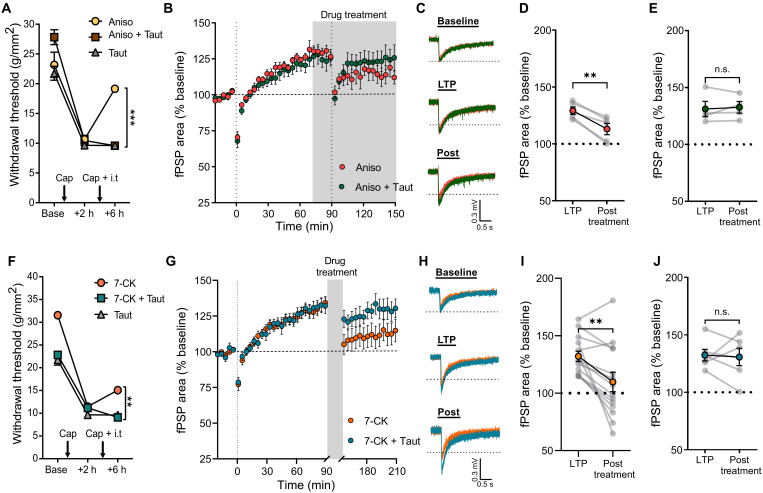
Anisomycin and 7-CK induce the reversal of hyperalgesia and dorsal horn LTP via PP1-dependent mechanism. (**A**) Intrathecal injection of tautomycetin (Taut; PP1 inhibitor) prevented the reversal of hyperalgesia by reactive destabilization. (**B**) After LTP induction, bath application of anisomycin (Aniso; red) or Aniso + Taut (green) at 75 min was followed by a second round of 2-Hz stimulation onto dorsal roots at 90 min. (**C**) Representative traces of fPSPs recorded at baseline, 90 min (LTP), and 150 min (Post). (**D** and **E**) fPSPs area compared before (LTP) and after (posttreatment) 2-Hz reactivation of potentiated pathways in the presence of (D) Aniso or (E) Aniso + Taut. (**F**) PP1 inhibition prevented the reversal of hyperalgesia by NI-NMDAR signaling. (**G**) Reversal of LTP by bath application of 7-CK (orange) and dorsal root stimulation was not observed when tautomycetin was applied with 7-CK (turquoise). (**H**) Representative traces of fPSPs recorded at baseline, 90 min (LTP), and 210 min (post). (**I** and **J**) fPSP area compared before (LTP) and after (posttreatment) administration of (I) 7-CK or (J) 7-CK + Taut. Data are means ± SEM. n.s., *P* > 0.05; **P < 0.01; ***P < 0.001.

The induction of synaptic long-term depression (LTD) by NI-NMDAR signaling is mediated by a conformational change in the C-terminal of the NMDAR GluN1 subunit, which allows protein phosphatase-1 (PP1) to access and dephosphorylate calcium/calmodulin-dependent protein kinase II (CaMKII) (*18*). The catalytic activity of PP1 is necessary for NI-NMDAR signaling to drive both functional and structural synaptic depression (*15*, *18*, *25*). We therefore sought to examine whether PP1 activity also contributes to the reversal of hyperalgesia by reactive destabilization. Pharmacological inhibition of PP1 with tautomycetin (5 μM, i.t.) prevented the reversal of hyperalgesia induced by the combination of anisomycin and intraplantar capsaicin (Fig. 3A). We further investigated the role of PP1 in reactive destabilization of dorsal horn LTP. We confirmed that bath application of anisomycin (100 μM) during the reactivation of potentiated pathways decreases the overall magnitude of dorsal horn LTP; however, this effect was prevented when tautomycetin (100 nM) was coapplied with anisomycin (Fig. 3, B to E), similar to our behavioral findings. In addition, we observed that the reversal of hyperalgesia by NI-NMDAR signaling also requires PP1 activity. Intrathecal administration of tautomycetin occluded the effects of 7-CK on hyperalgesia (Fig. 3F), while 7-CK–mediated reversal of LTP in spinal cord explants was prevented by bath application of tautomycetin (Fig. 3, G to J). In the downstream signaling of PP1, p38 mitogen-activated protein kinase (MAPK) activity is increased and required for LTD and spine shrinkage driven by NI-NMDAR signaling (*15*, *16*, *25*, *26*). Our in vivo results showed that p38 MAPK is required for both reactive destabilization and NI-NMDAR signaling. We found that SB-203580 (p38 MAPK inhibitor; 100 μM, i.t.) abolished the reversal of hyperalgesia mediated by anisomycin (fig. S6A) or 7-CK (fig. S6B). Overall, these data further indicate strong parallels between the mechanisms by which reactive destabilization and NI-NMDAR signaling reverse both mechanical hyperalgesia and synaptic potentiation in the dorsal horn.

### Ubiquitin-dependent proteasomal degradation of postsynaptic proteins underlies the depotentiation of sensitized pain pathways following reactive destabilization

Previous studies have shown that the degradation of postsynaptic proteins within the postsynaptic density (PSD), such as Shank and guanylate kinase associated protein (GKAP), is involved in the destabilization process after fear memory retrieval in the hippocampus (*27*) and in the amygdala (*28*). We therefore assessed whether the ubiquitin-proteasome pathway similarly contributes to the destabilization process after resensitization in the spinal cord.

**Fig. 4.**
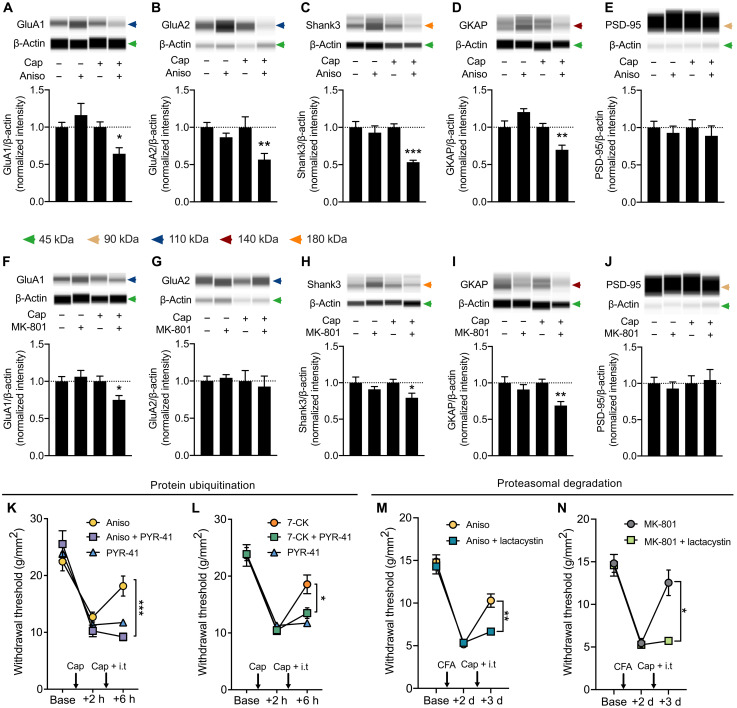
Ubiquitin-dependent proteosomal degradation of postsynaptic proteins underlies the depotentiation of sensitized pain pathways following destabilization. (**A** to **E**) CFA-induced hyperalgesia was reversed by induction of reactive destabilization via intrathecal injection of anisomycin and intraplantar capsaicin. Postsynaptic protein expression of (A) GluA1, (B) GluA2, (C) Shank3, (D) GKAP, and (E) PSD-95 in lumbar superficial dorsal horn tissue was quantified in animals treated with vehicle controls (Cap−, Aniso−), intrathecal anisomycin alone (Cap−, Aniso+), intraplantar capsaicin alone (Cap+, Aniso−), or treatment with both capsaicin and anisomycin (Cap+, Aniso+). (See also fig. S7, A to E). (**F** to **J**) CFA-induced hyperalgesia was similarly reversed by NI-NMDAR signaling, and postsynaptic protein in lumbar superficial dorsal horn tissue was quantified as described in (A) to (E). (See also fig. S7, F to J). (**K** to **N**) Requirement of ubiquitination and proteosome-mediated degradation to reverse hyperalgesia. Changes in mechanical withdrawal thresholds of capsaicin-induced hyperalgesia followed by a second ipsilateral intraplantar injection of capsaicin and intrathecal injection of (K) Aniso ± PYR-41 (ubiquitin-activating E1 enzyme inhibitor) and (L) 7-CK ± PYR-41. Changes in mechanical withdrawal thresholds of CFA-induced hyperalgesia followed by a second ipsilateral intraplantar injection of capsaicin and intrathecal injection of (M) Aniso ± lactacystin (protease inhibitor) and (N) MK-801 ± lactacystin. Data are means ± SEM. *P < 0.05; **P < 0.01; ***P < 0.001.

To examine whether the degradation of postsynaptic proteins at the SDH is induced by reactive destabilization or by NI-NMDAR signaling, we first performed immunoblot analyses with antibodies against five postsynaptic proteins in dorsal spinal cord tissue: GluA1, GluA2, Shank3, GKAP, and PSD-95. When anisomycin was intrathecally administered upon resensitization in CFA-treated mice, there was a significant reduction of protein expression in GluA1, GluA2, Shank3, and GKAP (Fig. 4, A to D). We did not observe changes in PSD-95 (Fig. 4E), implying that reactive destabilization does not induce a general loss of PSD or synapses. The down-regulation of specific postsynaptic proteins in the SDH following the induction of reactive destabilization suggests that this phenomenon involves an active process of synaptic destabilization to cause depotentiation, as previously speculated (*6*, *29*).

In addition, when NI-NMDAR signaling was induced via intrathecal MK-801 after resensitization, we found a pattern of altered protein expression similar to that seen with anisomycin. Here, we again observed reduced protein expression in GluA1 (Fig. 4F), Shank3, and GKAP (Fig. 4, H and I), with no change in PSD-95 (Fig. 4J). There was no change in protein levels of calcium-impermeable GluA2 subunit of the AMPA receptor (Fig. 4G). Notably, neither anisomycin nor MK-801 alone caused a change in the expression of these postsynaptic proteins.

Synaptic activity and memory recall can induce changes in synaptic proteins via the activation of the ubiquitin-proteasome pathway (*30*, *31*). We therefore assessed whether reactive destabilization and NI-NMDAR activity similarly involve the ubiquitin-proteasome system. A ubiquitin-activating E1 enzyme inhibitor, PYR-41 (135 μM), was administered together with either anisomycin (Fig. 4K) or 7-CK (Fig. 4L). PYR-41 prevented the reversal of hyperalgesia induced by both reactive destabilization and NI-NMDAR signaling, implying that protein ubiquitination is necessary in both processes. Because protein ubiquitination can mediate proteasomal degradation, we next asked whether selectively inhibiting proteasome activity would also prevent the reversal of hyperalgesia. Intrathecal administration of lactacystin (20 μM) also successfully abolished the reversal of hyperalgesia produced by either anisomycin (Fig. 4M) or MK-801 (Fig. 4N). Together, these results strongly indicate that the activity-dependent induction of the ubiquitin-proteasome system downstream of NI-NMDAR activity is responsible for the destabilization of sensitized pain pathway following reactivation.

## DISCUSSION

We used a sensory model of reconsolidation to investigate the cellular mechanisms by which encoded information in the nervous system can be dynamically changed. Our results reveal that the reactivation of sensitized nociceptive pathways triggered synaptic destabilization through NI-NMDAR signaling, enabling the reversal of central sensitization and pain sensitization. These results further demonstrate pivotal postsynaptic signaling mechanisms of the synaptic destabilization and degradation process that is fundamental for sensory reconsolidation.

Previous research into the mechanisms of reconsolidation has largely focused on defining the boundary and activation conditions that are necessary to induce reconsolidation and destabilize consolidated memories. Both memory and sensory reconsolidations have been shown to require activation of NMDARs (*32*–*34*). However, it was unclear which signaling mechanism was responsible for the destabilization process or whether NMDAR activity could directly engage destabilization. Our results demonstrated that the NI-NMDAR signaling pathway biases toward reactive destabilization and leads to synaptic weakening in the spinal cord. NMDAR activity is therefore necessary for both the synthesis and degradation of synaptic proteins in reconsolidation (*6*, *9*), making the NMDAR a pivotal signaling hub for this process.

The GluN2B subunit–containing NMDARs have been reported to drive the destabilization of memory traces, while GluN2A subunit–containing NMDARs support synaptic repotentiation and the reconsolidation of labile synapses (*35*, *36*). These observations suggest that NMDAR subunit composition plays a major role in the receptor’s ability to induce synaptic plasticity and regulate the stability of consolidated memories. Differences in the C-terminal interactions with intracellular effectors, for example, CaMKII, may explain the distinct roles of GluN2B and GluN2A in synaptic destabilization and repotentiation, respectively (*9*). Our study expands on this possibility by directly showing that C-terminal interactions of a completely different NMDAR subunit, the obligatory GluN1 subunit, can also drive the destabilization of sensitized pain pathways. It is notable that the developmental shift in the expression of NMDAR subtypes, in which dominant GluN2B expression gives way to GluN2A expression, does not occur in the SDH, leading to NMDAR responses dominated by GluN2B in adult dorsal horn neurons (*37*). The persistence of GluN2B may therefore facilitate the induction of NI-NMDAR activity and reactive destabilization in dorsal horn networks.

Labile memories require de novo protein synthesis to become stable, undergo reconsolidation, and persist over time (*38*). Upstream of protein synthesis, the repotentiation process of reconsolidation has been associated with translational control pathways involving CaMKII, protein kinase A (PKA), PKC, MAPK and mammalian target of rapamycin (mTOR), as well as multiple transcription factors including adenosine 3′,5′-monophosphate response element–binding protein, Zif268 and nuclear factor κB (*6*, *10*, *11*). However, much less is known about the depotentiation process that is also induced upon memory reactivation and underlies the reversal of hyperalgesia and dorsal horn LTP described here. Memory destabilization has been previously associated with the selective ubiquitination and degradation of GKAP and Shank scaffold proteins in the hippocampus and the amygdala (*27*, *28*). We observed that NI-NMDAR signaling–mediated reactive destabilization similarly involves ubiquitin-dependent proteasomal degradation, leading to the down-regulation of GKAP and Shank3 in the spinal cord dorsal horn.

We further observed that reactive destabilization and NI-NMDAR signaling had different effects on the expression of GluA2 subunit in the PSD, with GluA2 expression being lower after reactive destabilization than NI-NMDAR signaling. These discrepancies might be explained by the fact that these pharmacological inhibitors target different stages of the same signaling cascade. Anisomycin targets protein synthesis downstream of NMDAR activity without directly affecting receptor function; in the presence of anisomycin, both ionotropic and nonionotropic activities of NMDAR would engage upon receptor activation (*17*). Conversely, MK-801 isolates NI-NMDAR signaling by blocking Na^+^/Ca^2+^ cation influx through receptors, preventing the activation of calcium-sensitive protein kinases involved in synaptic plasticity (*39*). Postsynaptic Ca^2+^ influx has been shown to induce CaMKIV- and PKC-mediated endocytosis of the AMPA receptor GluA2 subunit (*40*, *41*), which may explain why GluA2 levels decrease in the presence of anisomycin but remain unchanged when cation influx is inhibited via MK-801. Nevertheless, NI-NMDAR signaling reduced both dorsal horn LTP and hyperalgesia, suggesting that this residual GluA2 capacity is insufficient to maintain sensitization.

NI-NMDAR signaling can be prevented by increased intracellular calcium influx mediated by postsynaptic voltage-gated calcium channels (*25*). Specifically, enhancing calcium influx through an increase in extracellular calcium concentration and administration of an L-type calcium channel agonist augmented signaling cascades normally initiated by ionotropic NMDAR activity and thus occluded calcium-independent NI-NMDAR signaling. These findings raise the possibility that pathological changes in calcium channel activity or expression could impede the endogenous activation of NI-NMDAR signaling. The expression of L-type calcium channel Ca_v_1.2 was increased in dorsal horn neurons in nerve injury neuropathic pain models (*42*, *43*). It remains to be determined whether targeting the expression or activity of voltage-gated calcium channels in nociceptive dorsal horn neurons would enable or enhance the resolution of pathological pain by endogenous NI-NMDAR signaling.

We additionally showed that NI-NMDAR signaling could reverse sensitization in different strains and sex of mice, suggesting that NI-NMDAR signaling may be a fundamental mechanism for the regulation of synaptic plasticity. This fundamental role would be consistent with the hypothesis that reconsolidation itself reflects an activity-dependent mechanism of homeostatic synaptic plasticity (*6*). The tools developed here to directly isolate NI-NMDAR signaling will enable further investigation of the endogenous role of NI-NMDAR signaling in plasticity and reconsolidation.

The ability of a memory trace to undergo reconsolidation depends on several factors, including the strength of initial reinforcement, the age of the memory, and prediction error during retrieval (*9*, *44*). Whether NI-NMDAR signaling and reactive destabilization of sensory plasticity will be similarly constrained by boundary conditions seen in memory destabilization and reconsolidation remains to be determined. These factors may affect the clinical significance of reactive destabilization for the treatment of chronic pain, including neuropathic pain that can persist for years and involve widespread changes in the central nervous system. Nevertheless, our results constitute a potential therapeutic indication that selective activation of NI-NMDAR signaling can reverse pathological pain, with the reversal of hypersensitivity persisting over days in long-lasting inflammatory pain models. It is notable that NI-NMDAR signaling had no effect on synaptic strength in the absence of LTP, suggesting that depotentiation is restricted to sensitized pathways. This would make NI-NMDAR signaling an ideal application for the treatment of pain without risk of altering normal sensory function.

## MATERIALS AND METHODS

### Experimental design

#### Animals

All behavioral experiments were conducted in accordance with the guidelines established by the Canadian Council for Animal Care and Local Animal Care Committee. Adult (>12-week-old) male C57BL/6N mice (Charles River, Sherbrooke, Quebec, Canada) were used for most experiments except indicated otherwise. Adult female C57BL/6N mice were used in Fig. 1 (C and F) and fig. S3 (E to G). Adult male CD-1 mice were used in fig. S3 (H to K). Mice were kept on a 14-hour light:10-hour dark cycle in groups of one to four mice per cage with food and water provided ad libitum. Animals were not reused in all behavioral experiments, and naïve mice were used in electrophysiological experiments.

#### Mechanosensitivity assay

All experiments were conducted on naïve mice and started before 10:00 a.m. Mechanosensitivity was measured using the SUDO up-down method with von Frey hairs to estimate the 50% withdrawal threshold in pressure units (in grams per square millimeter) (*45*). Care was taken to avoid the injection site when testing mechanosensitivity. Mechanical hyperalgesia was induced by intraplantar injection of capsaicin (5 μl, 0.5%, w/v). Inflammatory hyperalgesia was induced by intraplantar injection of CFA (10 μl), and all intraplantar and intrathecal injections (5 μl) were performed under light (<3 min) isoflurane anesthesia as previously described (*12*, *46*). In behavioral experiments, mice were excluded if they did not exhibit a reduction in withdrawal threshold greater than 10% after sensitization. Animals in which intrathecal injection did not produce an obvious tail flick were excluded from analysis. Animals were randomly assigned to treatment groups, and the experimenter was blinded during testing and data analysis. Because mechanical sensitivity can be altered by experimental conditions, such as the sex of the experimenter (*47*), all individual cohorts were tested by the same experimenter and under the same experimental conditions throughout the duration of the experiment. Behavioral data in Figs. 1I and 2 (E and F) and fig. S5 (B and D) were analyzed as percentage of maximum possible effect (MPE) using the formula 100% × [posttreatment paw withdrawal threshold (PWT) − pretreatment PWT] × (baseline PWT − pretreatment PWT)^−1^.

#### Electrophysiology

Electrophysiological recordings of dorsal root–evoked fPSPs were made using a whole spinal cord tissue preparation. Adult male C57BL/6N mice were anesthetized with chloral hydrate (400 mg/kg) and perfused with ice-cold sucrose-substituted artificial cerebrospinal fluid (sucrose aCSF; 50 mM sucrose, 92 mM NaCl, 15 mM d-glucose, 26 mM NaHCO_3_, 5 mM KCl, 1.25 mM NaH_2_PO_4_, 0.5 mM CaCl_2_, 7 mM MgCl_2_, and 1 mM kynurenic acid, bubbled with 95% oxygen:5% CO_2_). In some experiments, spinal explants were isolated from adult male CD-1 mice or adult female C57BL/6 N mice (Charles River). The lumbar spinal segment was removed and immersed in ice-cold sucrose aCSF, after which nervous tissue was quickly isolated via laminectomy. Ventral roots and connective tissue were removed from the spinal cord, and the tissue was placed in room-temperature aCSF (124 mM NaCl, 10 mM d-glucose, 26 mM NaHCO_3_, 3 mM KCl, 1.25 mM NaH_2_PO_4_, 2.6 mM CaCl_2_, and 1.3 mM MgCl_2_, bubbled with 95% oxygen:5% CO_2_) for 1 hour before experimentation. During experiments, the tissue was perfused with aCSF at room temperature at a flow rate of 6 to 8 ml/min.

fPSPs were recorded via a borosilicate glass electrode inserted into the dorsal side of the spinal cord at the dorsal root entry zone. Electrodes were inserted superficially to a depth of no more than 125 μm from the dorsal surface of the spinal cord, measured with an MPC-200 manipulator (Sutter Instrument Company, Novato, CA, USA). Electrodes had a tip resistance of 4 to 5 megohms when filled with aCSF. fPSPs were evoked by electrical stimulation of the dorsal root using a suction electrode that is pulled from borosilicate glass, filled with aCSF, and placed near the cut end of the dorsal root. Field potentials were amplified with a Multiclamp 700A amplifier (Molecular Devices, Sunnyvale, CA, USA), digitized with a Digidata 1322A digitizer (Molecular Devices), and recorded using pClamp 10 software (Molecular Devices). Data were filtered during acquisition with a low pass filter set at 2 kHz and sampled at 10 kHz.

Test stimuli were presented every 30 s to evoke fPSPs. The stimulus intensity was sufficient to activate C-fibers as indicated by the appearance of a third distinct fiber volley after the stimulus artifact, while a slightly (20%) higher intensity was used to induce LTP. LTP was induced by low-frequency stimulation (2 Hz for 2 min) of the dorsal root as described previously (*12*). This LTP likely reflects an increase in the net postsynaptic activation of superficial dorsal horn neurons since supraspinally projecting neurons only comprise a small percentage of the neurons in this area (*48*). After a stable baseline recording (20 min), the LTP protocol was presented at time = 0 min. Experimental drugs were added to the aCSF at 90 min and washed out at 150 min for a total application of 1 hour. For reconsolidation experiments, anisomycin and tautomycetin were added to the aCSF at 75 min, and a second round of 2-Hz stimulation was presented at time = 90 min. In experiments where LTP was not induced, 7-CK was administered at 0 min and washed out at 60 min. Data were analyzed using ClampFit 10 software (Molecular Devices). Representative electrophysiology traces are shown in Figs. 1K and 3 (C and H) and figs. S2 (B and E), S3B, and S4B. The area of fPSPs was measured from 0 to 800 ms after the onset of fPSPs. The averaged data from the complete dataset with analysis of complete data are shown in Figs. 1 (L and M) and 3 (D, E, I, and J); figs. S2 (C and F), S3 (C and D), and S4 (C, E, and F); and table S2. All drugs and chemicals for electrophysiology solutions were purchased from MilliporeSigma Canada (Oakville, ON, Canada).

#### NMDAR mimetic Tat peptide production

Cell permeable NMDAR mimetics C1.1 corresponding to amino acids 864 to 877 of GluN1 (DRKSGRAEPDPKKK), scrambled C1.1 (EPAKDDGRRKPSKK), C1.2 corresponding to amino acids 877 to 900 of GluN1 (KATFRAITSTLASS), and scrambled C1.2 (AATRSTFASKTLIS) were synthesized with a Tat sequence (YGRKKRRQRRR) at the C-terminal by GenScript (Piscataway, NJ, USA). Scrambled sequences were confirmed to not match other protein sequences in mice by Blast search.

#### Isolation of synaptic fractions

For biochemical studies, adult C57BL/6N mice were anesthetized with intraperitoneal injection of chloral hydrate (400 mg/kg; MilliporeSigma) and transcardiac perfused with ice-cold sucrose aCSF (50 mM sucrose, 92 mM NaCl, 15 mM d-glucose, 26 mM NaHCO_3_, 5 mM KCl, 1.25 mM NaH_2_PO_4_, 0.5 mM CaCl_2_, 7 mM MgCl_2_, and 1 mM kynurenic acid, bubbled with 95% oxygen:5% CO_2_). The spinal cord was removed from the lumbar spinal column via laminectomy under aCSF and immediately placed in ice-cold sucrose aCSF.

The lower lumbar region of the spinal cord was placed on a bed of dry ice/metal plate and allowed to freeze after which it was cut along the frontal plane to separate the dorsal horn section. Isolation of the synaptic fraction was performed as previously described, with minor modifications (*49*). Briefly, tissues were homogenized in microtube homogenizer (Bel-art) in 300 μl of lysis buffer [0.32 M sucrose and 5 mM Hepes (pH 7.4)] supplemented with one tablet of Roche Complete Mini-Protease Inhibitor (MilliporeSigma) and 100 μl of Phosphatase Inhibitor Cocktail 2 and 3 (MilliporeSigma). Aliquots of lysates were saved for further analyses as total homogenates. The rest of lysates went through two spins at 4°C (10 min at 1000*g* and 20 min at 12,000*g*) to obtain crude synaptic fractions. Supernatant containing the light membrane fraction and soluble enzymes was immediately frozen for further analyses as nonsynaptic fraction, and pellet containing crude synaptosome was resuspended in 200 μl of 0.01 M phosphate-buffered saline. Successful subcellular fraction was confirmed by Western blot (fig. S8), and protein concentration was measured using Pierce BCA protein Assay Kit (Thermo Fisher Scientific, Mississauga, Ontario, Canada). Samples were stored at −80°C until use.

#### Western blotting

Protein expression was assessed using the Wes capillary electrophoresis system (Protein Simple, San Jose, CA, USA) according to the manufacturer’s manual. Briefly, samples were diluted to 0.1 μg/μl in provided 1× sample buffer and 1× fluorescent molecular weight marker/reducing agent (Protein Simple). Samples were then vortexed and heat-denatured for 5 min at 95°C. Samples (0.3 μg) were loaded to each lane into the Wes assay plate (Protein Simple), and 12- to 230-kDa separation modules were used. Proteins were separated using capillary electrophoresis and probed with each of the following primary antibodies under default run conditions: anti-GluA1 (1:100; ab109450, Abcam), anti-GluA2 (1:400; ab206293, Abcam), anti-GKAP (1:100; CST #13602. Cell Signaling Technologies, Danvers, MA, USA), anti-Shank3 (1:400; CST #64555, Cell Signaling Technologies), and anti–PSD-95 (1:100; CST #3450, Cell Signaling Technologies). Protein expression was normalized to the internal control β-actin (1:100; CST #4970, Cell Signaling Technologies). Both primary antibodies for target protein and loading control were premixed before loading into the Wes assay plate according to the manufacturer’s protocol. The dilutions of primary antibodies have been optimized and validated to ensure sufficient saturation of the protein bound to the capillary wall. The band density measured using the Wes system was within a functional dynamic range for quantitative comparisons of signal between samples. Accordingly, the exposure time was set to sufficiently show both target protein and loading protein on the same blot. Protein was detected using the anti-rabbit detection kit (#DM-001, Protein Simple). Protein was quantified by area under the curve of the chemiluminescent signal. For Western blot data, the averaged data of normalized bands for each treatment group with complete analysis are also shown in Fig. 4 (A to J) and table S2. Data were visualized and analyzed using Compass software version 4.0 for Simple Western (Protein Simple). Representative Western blot images are shown in Fig. 4 (A to J). Full-length Western blot images are also available in fig. S7.

#### Quantification and statistical analysis

The criterion for exclusion was established before experiments. In behavioral experiments, mice were excluded if they did not exhibit a reduction in withdrawal threshold greater than 10% after sensitization. Animals in which intrathecal injection did not produce an obvious tail flick were excluded from analysis. Animals were randomly assigned to experimental groups in all experiments using a counterbalanced approach. The experimenter was blinded to experimental groups during testing and analyzing data.

Statistical analyses were performed using GraphPad Prism v9. In all figures, results are expressed as the means ± SEM. All tests were two-sided. MPE was compared between groups using a one-way analysis of variance (ANOVA), followed by Tukey’s multiple comparison post hoc test or Student’s *t* test as appropriate. Withdrawal thresholds in the single capsaicin injection experiment, and CFA hyperalgesia were compared on each day and time point, respectively, using a two-way repeated-measures ANOVA with Bonferroni post hoc test. In biochemical analyses, results were analyzed statistically using one-way ANOVA to the vehicle group, followed by a post hoc Holm-Sidak test.

Average fPSPs area was compared between experimental groups using paired Student’s *t* tests. No sample size calculation was predetermined. Samples sizes used reflect a balance between sample sizes generally used in the field for statistical power and to minimize the use of animals in pain experiments where possible. A statistics table for each figure is available in table S2.

## Notes

View/request a protocol for this paper from *Bio-protocol*.
