# Supplementary material for: Nociceptor activity induces nonionotropic NMDA receptor signaling to enable spinal reconsolidation and reverse pathological pain
Source: Sci Adv. 2023 May 19;9(20):eadg2819. doi: 10.1126/sciadv.adg2819 (PMC10198626; doi:10.1126/sciadv.adg2819)
Supplement: Supplementary file 1 — Figs. S1 to S8 Tables S1 and S2 [file sciadv.adg2819_sm.pdf]

Science Advances
AAAS

Supplementary Materials for

Nociceptor activity induces nonionotropic NMDA receptor signaling to enable spinal reconsolidation and reverse pathological pain

Hantao Zhang *et al.*

Corresponding author: Robert P. Bonin, rob.bonin@utoronto.ca

Sci. Adv. **9**, eadg2819 (2023)
DOI: 10.1126/sciadv.adg2819

This PDF file includes:

Figs. S1 to S8
Tables S1 and S2

Figure S1

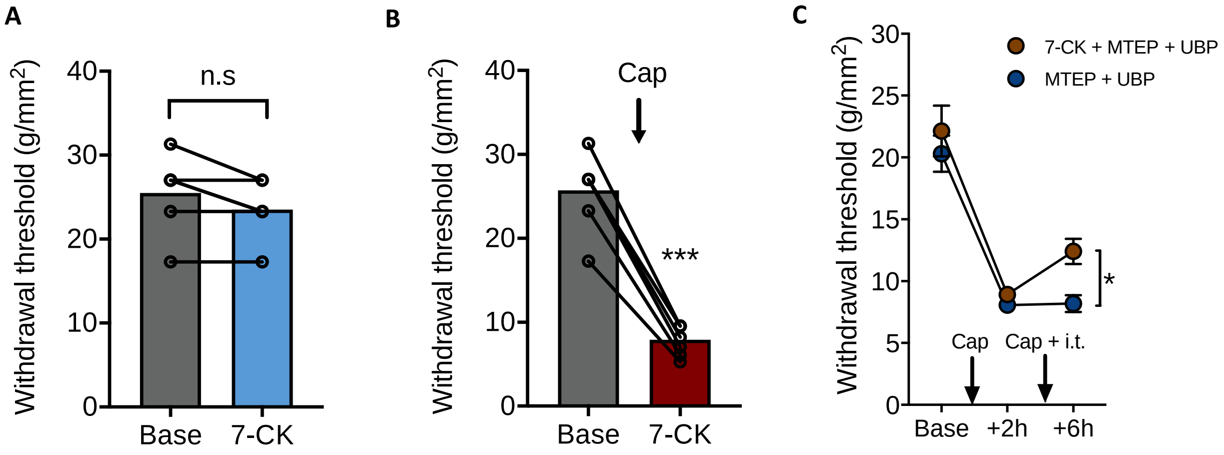

Figure S1. NI-NMDAR activity does not affect baseline mechanical sensitivity, it does not supress pain processing in naïve C57BL/6N mice, and it is independent of GluK1 and mGluR5 receptors. Changes in mechanical withdrawal thresholds after: (A) intrathecal injection of 7-CK, (B) intrathecal injection of 7-CK followed by intraplantar capsaicin injection, or (C) intrathecal injection of MTEP hydrochloride (MTEP, mGluR5 receptor antagonist) + UBP-310 (UBP, GluK1 receptor antagonist) ± 7-CK at the time of re-sensitization. Data are mean ± SEM. n.s. $p > 0.05$; $^{*}p < 0.05$; $^{***}p < 0.001$.

Figure S2

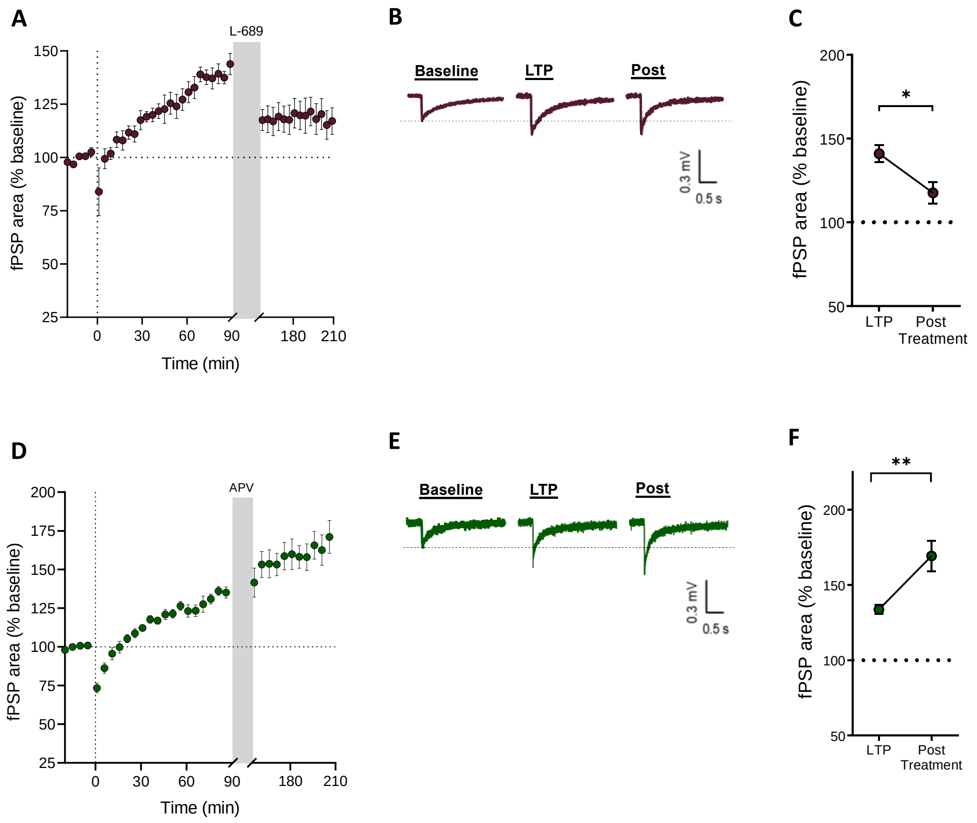

Figure S2. A different NMDAR glycine site antagonist also reverses dorsal horn LTP, while glutamate site antagonist is unable to decrease LTP magnitude.

(A) After LTP induction, bath application of glycine site antagonist (L-689,560) from 90min to 150min, was coupled with electrical stimulation of dorsal roots. (B) Representative traces of fPSPs recorded at baseline, 90min (LTP) and 210min (Post). (C) fPSPs area compared before (LTP) and after (Post Treatment) L-689,560 administration. (D) Bath application of NMDAR glutamate site antagonist (APV) during reactivation of sensitized pathways does not reverse dorsal horn LTP. (E) Representative traces of fPSPs recorded at baseline, 90min (LTP) and 210min (Post). (F) fPSPs area compared before (LTP) and after (Post Treatment) APV administration. Data are mean ± SEM. *$p < 0.05$; **$p < 0.01$.

Figure S3

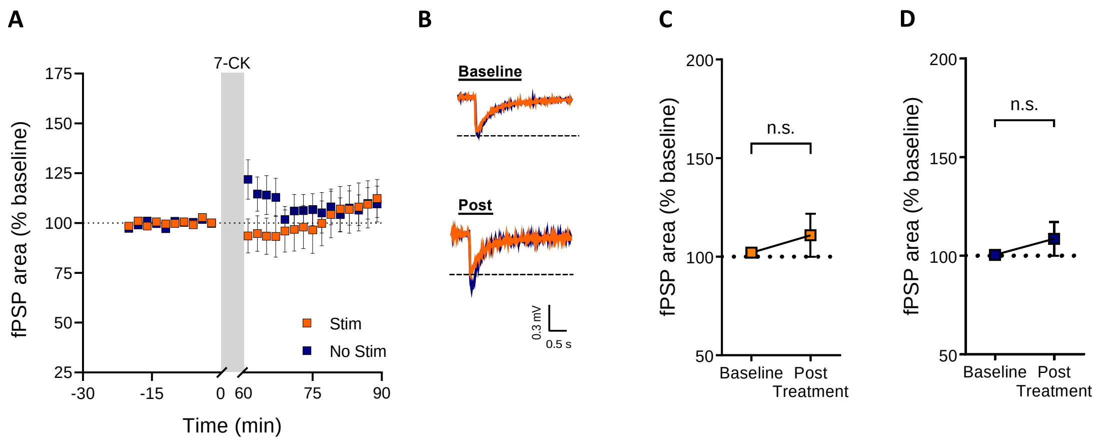

Figure S3. NI-NMDAR activity does not affect dorsal horn excitability without prior induction of LTP.

(A) After a stable baseline, 7-CK was bath applied from 0 min to 60 min, without prior induction of LTP. Dorsal roots were electrically stimulated (Orange; Stim) or left unstimulated (Blue; No Stim) during 7-CK administration. (B) Representative traces of fPSPs recorded at baseline and 90 min (Post). (C, D) fPSPs area compared before (Baseline) and after (Post Treatment) 7-CK administration (C) with stimulation or (D) without stimulation. Data are mean ± SEM. n.s. indicates $p > 0.05$.

Figure S4

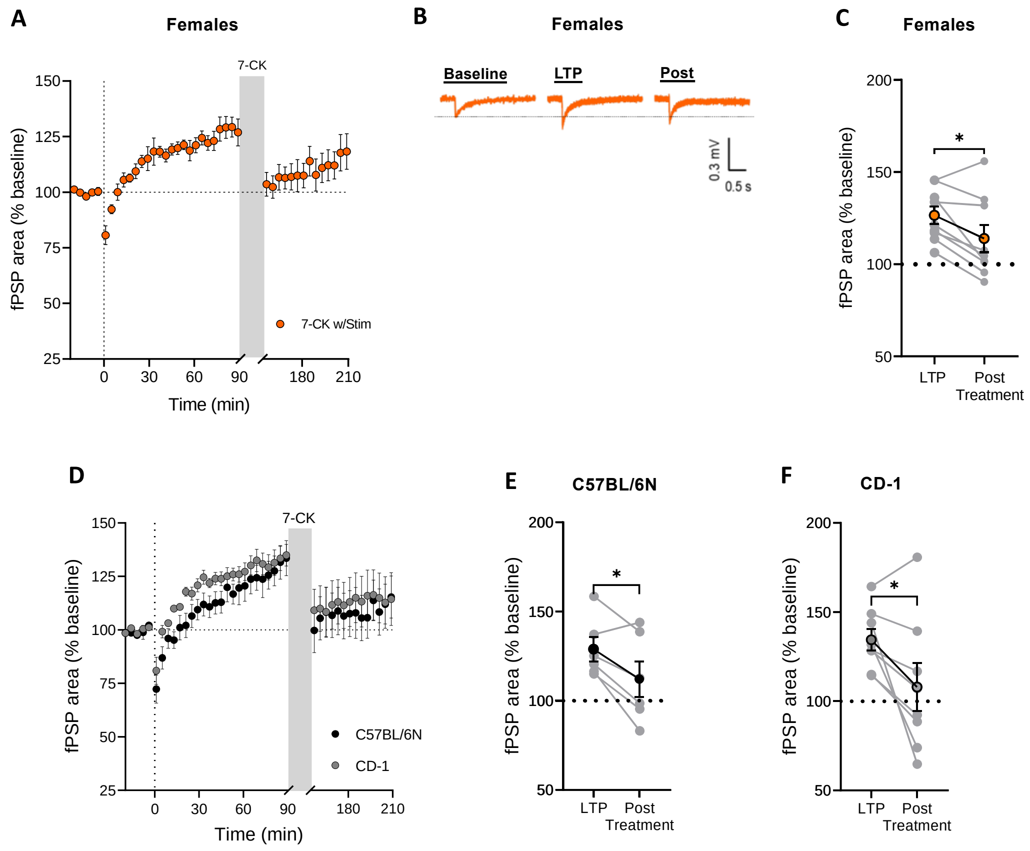

Figure S4. NI-NMDAR activity can reverse dorsal horn LTP in both sexes, and across two common strains of laboratory mice.

In female C57BL/6N mice: (A) LTP of fPSPs in the SDH was induced via 2Hz stimulation of dorsal roots followed by bath application of 7-CK from 90min to 150mins with electrical stimulation of sensitized pathways. (B) Representative traces of fPSPs recorded at baseline, 90min (LTP) and 210min (Post). (C) fPSPs area compared before (LTP) and after (Post Treatment) 7-CK application with stimulation of dorsal roots. (D) NI-NMDAR activity reversed dorsal horn LTP in C57BL/6N mice (Black) and CD-1 mice (Grey). (E, F) Comparison of fPSPs area before (LTP) and after (Post Treatment) 7-CK administration with stimulation in (E) C57BL/6N mice, or (F) CD-1 mice. Data are mean ± SEM. *$p < 0.05$.

Figure S5

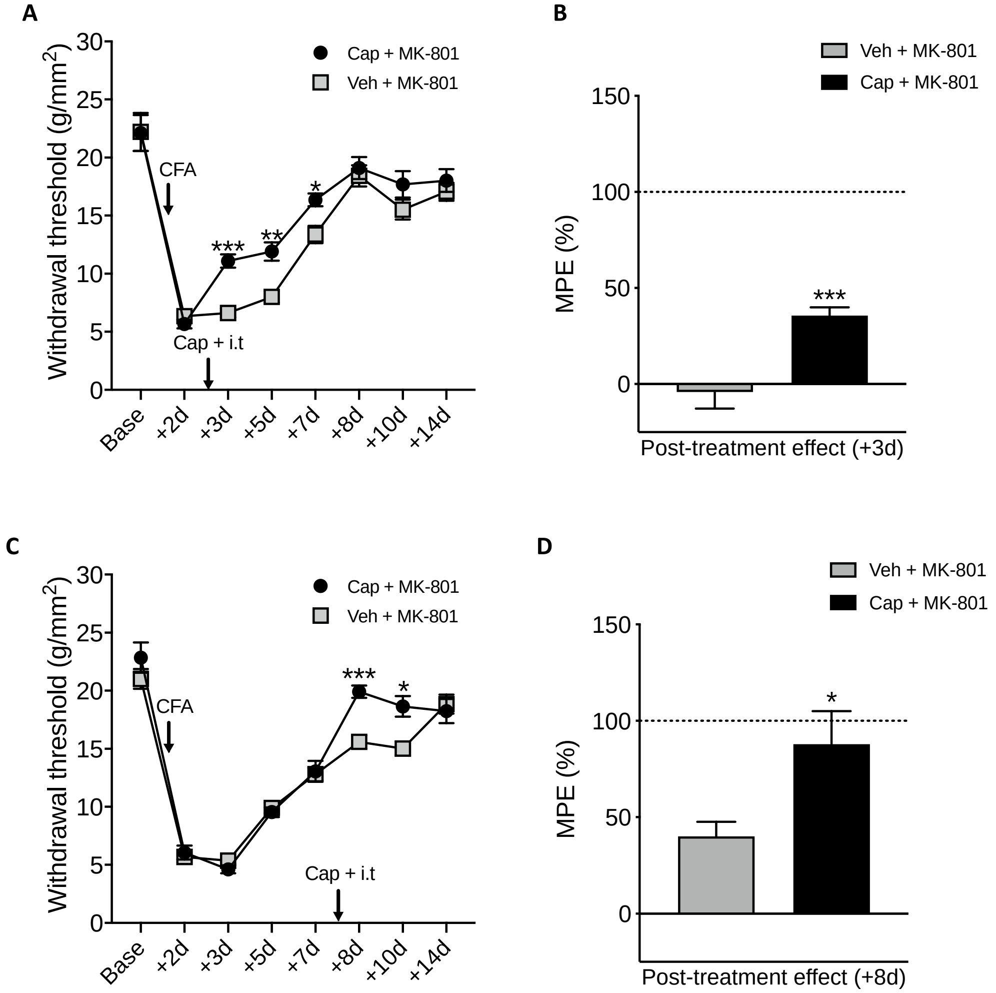

Figure S5. NI-NMDAR activity produces long-lasting analgesic effects and reduces recovery time from CFA-induced hyperalgesia.

(A) Changes in mechanical withdrawal thresholds induced by intraplantar Complete Freund's Adjuvant (CFA) followed by a second ipsilateral intraplantar injection of capsaicin (Cap) or vehicle (Veh) and i.t. injection of MK-801 on day 2 (n = 12 mice per group). (B) Summary of antihyperalgesia effect in (A) on day 3 (i.e.: 24 hours after treatment) expressed as percentage of Maximum Possible Effect (MPE). (C) Changes in mechanical withdrawal thresholds induced by intraplantar Complete Freund's Adjuvant (CFA) followed by a second ipsilateral intraplantar injection of capsaicin (Cap) or vehicle (Veh) and i.t. injection of MK-801 on day 7 (n = 12 mice per group). (D) Summary of antihyperalgesia effect in (C) on day 8 (i.e.: 24 hours after treatment) expressed as percentage of Maximum Possible Effect (MPE). The approximate time of the injections in (A) and (C) are indicated by arrows. All data are presented as mean ± SEM. *$p<0.05$; **$p < 0.01$; ***$p < 0.001$.

Figure S6

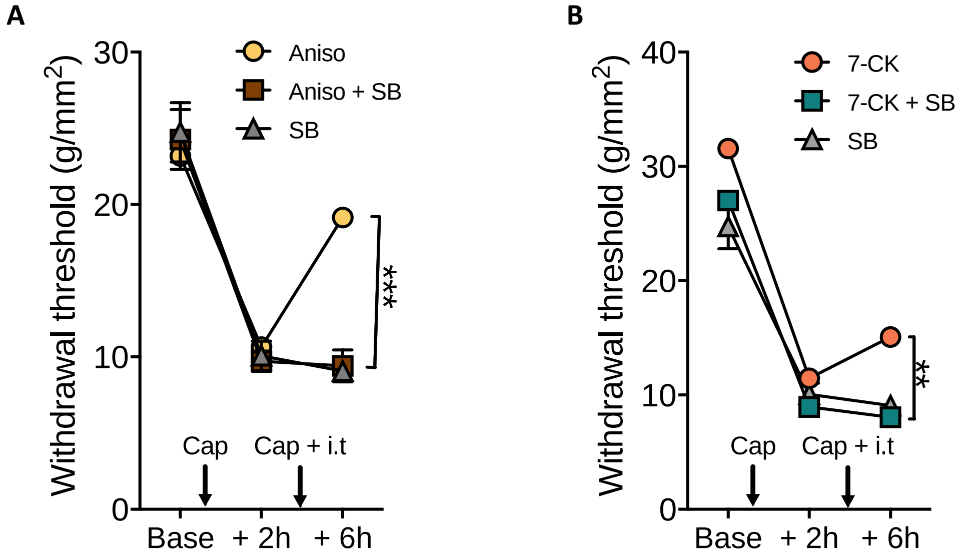

Figure S6. Both reactive destabilization and NI-NMDAR signaling are dependent on p38 MAPK activation.

Changes in mechanical withdrawal thresholds induced by intraplantar injection of capsaicin (Cap) followed by a second ipsilateral intraplantar injection of capsaicin (Cap) and intrathecal injection (i.t.) of: (A) anisomycin (Aniso) ± SB-203580 (SB, p38 MAPK inhibitor), or (B) 7-CK ± SB. Controls with i.t. SB alone shown in both (A) and (B). Data are mean ± SEM. **$p < 0.01$; ***$p < 0.001$.

Figure S7

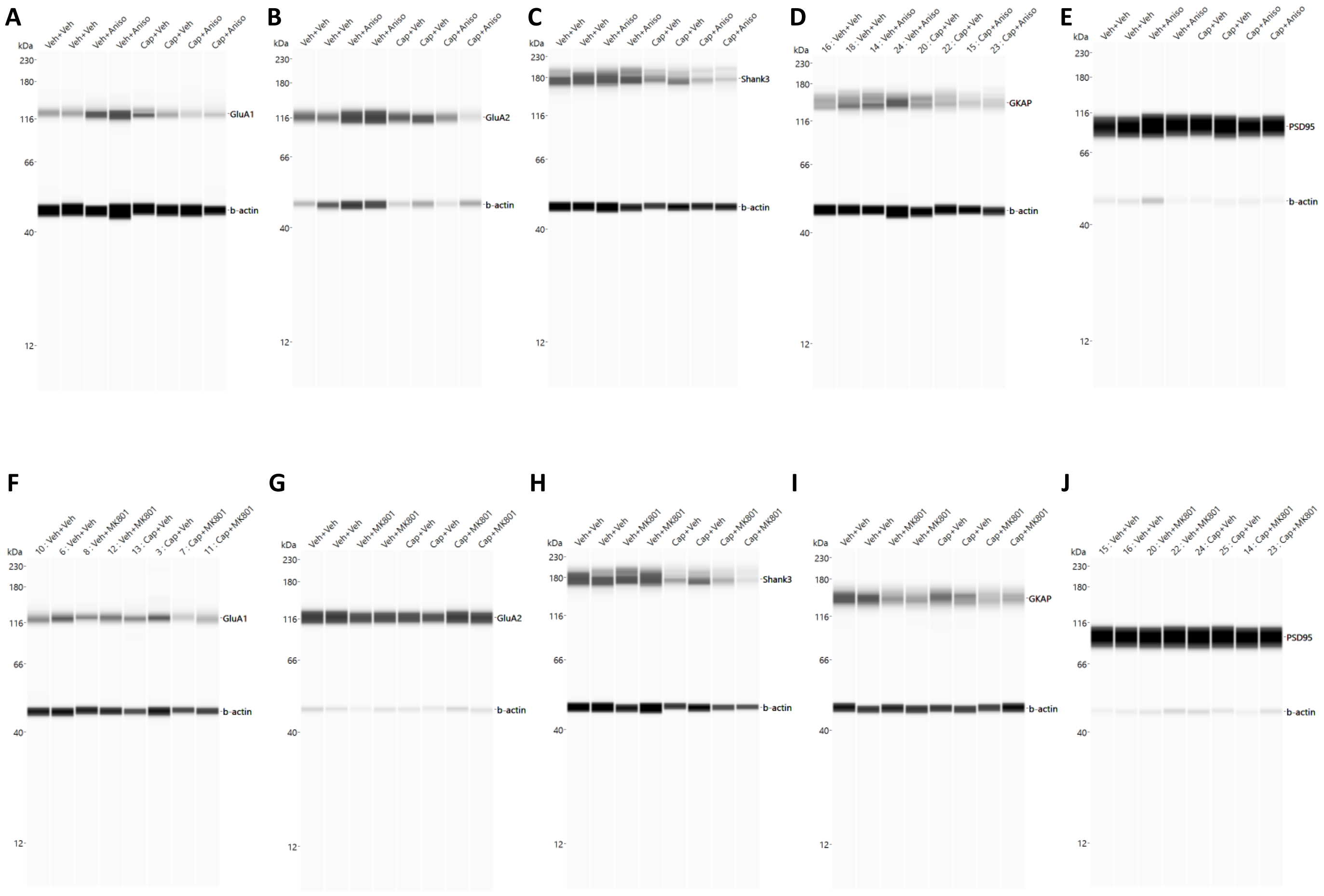

Figure S7. Full-length Western blot images.

Full-length Western blot image for (A) Figure 4A; (B) Figure 4B; (C) Figure 4C; (D) Figure 4D; (E) Figure 4E; (F) Figure 4F; (G) Figure 4G; (H) Figure 4H; (I) Figure 4I; (J) Figure 4J.

Figure S8

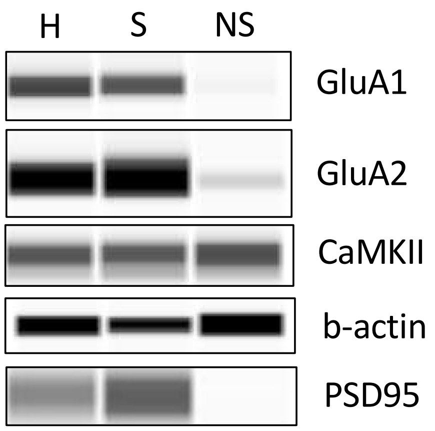

Figure S8. Representative western blot of total homogenate and subcellular fraction protein from mouse spinal cord.

Synaptic protein expression in different spinal dorsal horn protein fractions; H = homogenized tissue, S = synaptosome fraction, NS = non-synaptic fraction. Western blot analysis of crude synaptosome preparation showed that synaptic markers were strongly maintained in crude synaptosome fraction. Immunoblots of total homogenate (H), crude synaptosome (S), and cytosol non-synaptic fraction (NS) were probed for GluA1 (postsynaptic membrane), GluA2 (postsynaptic membrane), CaMKII (synaptic), b-actin (structural, loading control), and PSD95 (postsynaptic density).

Table S1. Key reagents/resources table

REAGENT or RESOURCE	SOURCE	IDENTIFIER
Antibodies		
Rabbit Monoclonal anti-GluA1	Abcam	Cat# ab109450
Rabbit Monoclonal anti-GluA2	Abcam	Cat# ab206293
Rabbit Polyclonal anti-GKAP	Cell signaling technology	Cat# CST 13602
Rabbit Monoclonal anti-Shank3	Cell signaling technology	Cat# CST 64555
Rabbit Monoclonal anti-PSD95	Cell signaling technology	Cat# CST 3450
Rabbit Monoclonal anti- β -actin	Cell signaling technology	Cat# CST 4970
Chemicals, and peptides		
Anisomycin	Cayman Chemical	Cat# 11308
7-Chlorokynurenic acid (7-CK)	Abcam	Cat# ab120024
APV	Millipore-Sigma Canada	Cat# A8054
NMDA	Millipore-Sigma Canada	Cat# M3262
Capsaicin	Millipore-Sigma Canada	Cat# M2028
L-689,560	Alomone Labs	Cat# L225
AV-101	Vistagen	N/A
Complete Freund's Adjuvant	Millipore-Sigma Canada	Cat# F5881
(+)-MK-801 maleate	Tocris Biotechne	Cat# 0924
Tautomycetin	Tocris Biotechne	Cat# 2305
PYR-41	Tocris Biotechne	Cat# 2978
Lactacystin	Tocris Biotechne	Cat# 2267
MTEP hydrochloride	Tocris Biotechne	Cat# 2921
UBP-310	Tocris Biotechne	Cat# 3621
SB-203580	Tocris Biotechne	Cat# 1202
TAT-NMDAR$_{C1.1}$[864-877]: (YGRKKRRQRRR**DRKSGRAEPDPKKK**)	GenScript	Custom Synthesis
TAT-NMDAR$_{C1.2}$[877-900]:	GenScript	Custom Synthesis

(YGRKKRRQRRR**KATFRAITSTLASS**)		
TAT-NMDAR$_{Scrambled\ C1.1}$: (YGRKKRRQRRR**EPAKDDGRRKPSKK**)	GenScript	Custom Synthesis
TAT-NMDAR$_{Scrambled\ C1.2}$: (YGRKKRRQRRR**AATRSTFASKTLIS**)	GenScript	Custom Synthesis
Experimental Models: Organisms/Strain		
C57BL/6N	Charles River Laboratories	N/A
CD-1	Charles River laboratories	N/A
Software and algorithm		
Axon pCLAMP 10	Molecular Devices	https://mdc.custhelp.com/euf/assets/content/pCLAMP10_User_Guide.pdf
Graphpad Prism 8	Graphpad	https://www.graphpad.com/

Table S2. Statistics Table

	Test Used		n		Descriptive Stats (Average, variance)	P Value	Degree of freedom & F/t/z/R/ETC Value
Figure Number	Test	Section	Exact value	Defined	Reported	Exact Value	Exact Value
1B	2-way RM-ANOVA	Fig 1	10,10,10	mice	mean ± SEM	P <0.001	F(4,54) = 9.6
1C	2-way RM-ANOVA	Fig 1	10,10,10	mice	mean ± SEM	P<0.001	F(4,54) = 16.7
1D	2-way RM-ANOVA	Fig 1	6,6,12	mice	mean ± SEM	P<0.001	F(2,21) = 14.0
1E	2-way RM-ANOVA	Fig 1	6,6,6	mice	mean ± SEM	p = 0.014	F(2,15) = 5.7
1F	2-way RM-ANOVA	Fig 1	7,7	mice	mean ± SEM	p = 0.011	F(2,24) = 5.4
1G	2-way RM-ANOVA	Fig 1	8,7	mice	mean ± SEM	p < 0.001	F (1,13) = 20.7
1H	2-way RM-ANOVA	Fig 1	4,4	mice	mean ± SEM	p = 0.017	F(2,12) = 5.8
1I	1-way ANOVA	Fig 1	4,4,4,4	mice	mean ± SEM	p = 0.0016	F(3,12) = 9.7
1L	paired t-test	Fig 1	14,14	mice	mean ± SEM	p = 0.0038	t(13) = 3.5
1M	paired t-test	Fig 1	6,6	mice	mean ± SEM	p = 0.68	t(5) = 0.40
2B	2-way RM-ANOVA	Fig 2	6,6	mice	mean ± SEM	p<0.001	F(2,20) = 131.1

2C	2-way RM-ANOVA	Fig 2	6,6	mice	mean ± SEM	p < 0.001	F(2, 20) = 10.9
2E	1-way ANOVA	Fig 2	7,9,9,7,7	mice	mean ± SEM	p < 0.001	F(4,34) = 7.2
2F	1-way ANOVA	Fig 2	16,10,10, 7,7	mice	mean ± SEM	p < 0.001	F(4,45) = 7.2
2G	2-way RM-ANOVA	Fig 2	6,5,5	mice	mean ± SEM	p = 0.19	F(2,13) = 1.9
3A	2-way RM-ANOVA	Fig 3	6,6,6	mice	mean ± SEM	p < 0.001	F(2,25) = 11.0
3D	paired t-test	Fig 3	5,5	mice	mean ± SEM	p = 0.0035	t(13) = 3.5
3E	paired t-test	Fig 3	4,4	mice	mean ± SEM	p = 0.67	t(3) = 0.5
3F	2-way RM-ANOVA	Fig 3	6,6,6	mice	mean ± SEM	p < 0.001	F(2,25) = 11.1
3I	paired t-test	Fig 3	14,14	mice	mean ± SEM	p = 0.0038	t(13) = 3.5
3J	paired t-test	Fig 3	6,6	mice	mean ± SEM	p = 0.82	t(5) = 0.2
4A	1-way ANOVA	Fig 4	8,8,8,8	mice	mean ± SEM	p = 0.0090	F (3,28) = 4.7
4B	1-way ANOVA	Fig 4	8,8,8,8	mice	mean ± SEM	p = 0.0070	F (3,28) = 4.9
4C	1-way ANOVA	Fig 4	7,8,8,8	mice	mean ± SEM	p < 0.001	F (3,27) = 12.1
4D	1-way ANOVA	Fig 4	8,8,8,8	mice	mean ± SEM	p < 0.001	F (3, 28) = 11.3
4E	1-way ANOVA	Fig 4	8,8,8,8	mice	mean ± SEM	p = 0.84	F (3, 28) = 0.3
4F	1-way ANOVA	Fig 4	8,8,8,8	mice	mean ± SEM	p = 0.021	F (3,28) = 3.8
4G	1-way ANOVA	Fig 4	8,8,8,8	mice	mean ± SEM	p = 0.89	F (3,28) = 0.2

4H	1-way ANOVA	Fig 4	7,8,8,8	mice	mean ± SEM	$p = 0.050$	$F (3, 27) = 2.9$
4I	1-way ANOVA	Fig 4	8,8,8,8	mice	mean ± SEM	$p = 0.0060$	$F (3,28) = 5.1$
4J	1-way ANOVA	Fig 4	8,8,8,8	mice	mean ± SEM	$p = 0.90$	$F (3,28) = 0.2$
4K	2-way RM-ANOVA	Fig 4	6,6,16	mice	mean ± SEM	$p = 0.0010$	$F(4,50) = 5.3$
4L	2-way RM-ANOVA	Fig 4	6,14,16	mice	mean ± SEM	$p = 0.010$	$F(4,66) = 3.6$
4M	2-way RM-ANOVA	Fig 4	9,8	mice	mean ± SEM	$p = 0.0050$	$F(2,30) = 6.2$
4N	2-way RM-ANOVA	Fig 4	9,7	mice	mean ± SEM	$p = 0.0020$	$F(2,28) = 7.5$
S1A	paired t-test	Fig S1	6,6	mice	mean ± SEM	$p = 0.077$	$t(5) = 2.2$
S1B	paired t-test	Fig S1	7,7	mice	mean ± SEM	$p < 0.001$	$t(6) = 15.5$
S1C	2-way RM-ANOVA	Fig S1	6,6	mice	mean ± SEM	$p = 0.0090$	$F (1,10) = 10.5$
S2C	paired t-test	Fig S2	7,7	mice	mean ± SEM	$p = 0.029$	$t(6) = 2.8$
S2F	paired t-test	Fig S2	7,7	mice	mean ± SEM	$p = 0.0069$	$t(6)= 4.0$
S3C	paired t-test	Fig S3	9,9	mice	mean ± SEM	$p = 0.39$	$t(8) = 0.9$
S3D	paired t-test	Fig S3	11,11	mice	mean ± SEM	$p = 0.34$	$t(10) = 1.0$
S4C	paired t-test	Fig S4	9,9	mice	mean ± SEM	$p = 0.015$	$t(8) = 3.1$
S4E	paired t-test	Fig S4	6,6	mice	mean ± SEM	$p = 0.028$	$t(5) = 3.1$
S4F	paired t-test	Fig S4	8,8	mice	mean ± SEM	$p = 0.039$	$t(7) = 2.5$

S5A	2-way RM-ANOVA	Fig S5	12,12	mice	mean ± SEM	p = 0.034	F(7,154) = 2.2
S5B	unpaired t-test	Fig S5	12,12	mice	mean ± SEM	p < 0.001	t(22) = 4.1
S5C	2-way RM-ANOVA	Fig S5	12,12	mice	mean ± SEM	p < 0.001	F(7,154) = 4.2
S5D	unpaired t-test	Fig S5	12,12	mice	mean ± SEM	p = 0.018	t(22) = 2.6
S6A	2-way RM-ANOVA	Fig S6	8,7,6	mice	mean ± SEM	P < 0.001	F(2,30) = 7.4
S6B	2-way RM-ANOVA	Fig S6	8,7,6	mice	mean ± SEM	p = 0.0012	F(2,30) = 5.6
